# Comprehensive characterization of immune landscape of Indian and Western triple negative breast cancers

**DOI:** 10.1016/j.tranon.2022.101511

**Published:** 2022-08-11

**Authors:** Aruna Korlimarla, Hari PS, Jyoti Prabhu, Chanthirika Ragulan, Yatish Patil, Snijesh VP, Krisha Desai, Aju Mathews, Sandhya Appachu, Ravi B. Diwakar, Srinath BS, Alan Melcher, Maggie Cheang, Anguraj Sadanandam

**Affiliations:** a St. John's Research Institute, St. John's National Academy of Health Sciences, Bangalore, India; b Sri Shankara Cancer Hospital and Research Centre, Bangalore, India; c Division of Molecular Pathology, The Institute of Cancer Research, London, UK; d MOSC Medical College, Kolenchery, Kerala, India; e Centre for Translational Immunotherapy, Division of Radiotherapy and Imaging, The Institute of Cancer Research, London, UK; f Clinical Trials and Statistical Unit, The Institute of Cancer Research, London, UK; g Centre for Global Oncology, Division of Molecular Pathology, The Institute of Cancer Research, 15 Cotswold Road, Sutton, London SM2 5NG, UK

**Keywords:** Triple-negative breast cancer, Immunotherapy, Immune subtypes, Immune cells, Global oncology, India, TNBC, Triple-negative breast cancer, TIL, Tumor-infiltrating lymphocytes, Th, T-helper cells, TCR, T-cell receptor, DAMP, Damage-associated molecular pattern, ER, Estrogen receptor, PR, Progesterone receptor, HER2, Human epidermal growth factor receptor 2, US, United States, H&E, Hematoxylin and eosin, FFPE, Formalin-fixed paraffin embedded, ML, Machine learning, PPCCA, Probabilistic principal component and covariate analysis, IHC, Immunohistochemistry, SAM, Significance analysis of microarrays, PAM, Prediction analysis for microarrays, TCGA, The Cancer Genome Atlas, METABRIC, Molecular Taxonomy of Breast Cancer International Consortium, OS, Overall survival, ssGSEA, Single-sample geneset enrichment analysis, MHC, major histocompatibility class, APC, Antigen Presenting Cells, APP, Antigen processing and presentation, TAP, Transporter associated with antigen presenting, HLA, Human leukocyte antigen, BCR, B cell receptor, pPC, probabilisitic prinicipal component analysis, RPPA, Reverse phase protein array

## Abstract

**Purpose:**

Triple-negative breast cancer (TNBC) is a heterogeneous disease with a significant challenge to effectively manage in the clinic worldwide. Immunotherapy may be beneficial to TNBC patients if responders can be effectively identified. Here we sought to elucidate the immune landscape of TNBCs by stratifying patients into immune-specific subtypes (immunotypes) to decipher the molecular and cellular presentations and signaling events of this heterogeneous disease and associating them with their clinical outcomes and potential treatment options.

**Experimental Design:**

We profiled 730 immune genes in 88 retrospective Indian TNBC samples using the NanoString platform, established immunotypes using non-negative matrix factorization-based machine learning approach, and validated them using Western TNBCs (*n*=422; public datasets). Immunotype-specific gene signatures were associated with clinicopathological features, immune cell types, biological pathways, acute/chronic inflammatory responses, and immunogenic cell death processes. Responses to different immunotherapies associated with TNBC immunotypes were assessed using cross-cancer comparison to melanoma (*n*=504). Tumor-infiltrating lymphocytes (TILs) and pan-macrophage spatial marker expression were evaluated.

**Results:**

We identified three robust transcriptome-based immunotypes in both Indian and Western TNBCs in similar proportions. Immunotype-1 tumors, mainly representing well-known claudin-low and immunomodulatory subgroups, harbored dense TIL infiltrates and T-helper-1 (Th1) response profiles associated with smaller tumors, pre-menopausal status, and a better prognosis. They displayed a cascade of events, including acute inflammation, damage-associated molecular patterns, T-cell receptor-related and chemokine-specific signaling, antigen presentation, and viral-mimicry pathways. On the other hand, immunotype-2 was enriched for Th2/Th17 responses, CD4^+^ regulatory cells, basal-like/mesenchymal immunotypes, and an intermediate prognosis. In contrast to the two T-cell enriched immunotypes, immunotype-3 patients expressed innate immune genes/proteins, including those representing myeloid infiltrations (validated by spatial immunohistochemistry), and had poor survival. Remarkably, a cross-cancer comparison analysis revealed the association of immunotype-1 with responses to anti-PD-L1 and MAGEA3 immunotherapies.

**Conclusion:**

Overall, the TNBC immunotypes identified in TNBCs reveal different prognoses, immune infiltrations, signaling, acute/chronic inflammation leading to immunogenic cell death of cancer cells, and potentially distinct responses to immunotherapies. The overlap in immune characteristics in Indian and Western TNBCs suggests similar efficiency of immunotherapy in both populations if strategies to select patients according to immunotypes can be further optimized and implemented.

## Highlights

• By comprehensively profiling the immune transcriptome of triple-negative breast cancers (TNBCs) and applying machine learning methods, we identified three immunotypes (1, 2 and 3) with significant differences in prognosis and therapy responses.
• TNBC is much more common in India than in the West. However, there was no significant difference in immune transcriptome between Indian and Western TNBCs in our study.
• Our analysis shows biological, signaling and clinical features of three TNBC immunotypes.
• Immunotype-1 gene signature is associated with improved prognosis and treatment responses in a cross-cancer comparison analysis of melanoma patients treated with anti-PDL1 therapy.
• Our study identified a potential opportunity to stratify patients for MAGEA3 and anti-PDL1-based therapies, which warrants further validation.

## Introduction

Breast cancer is the foremost cause of cancer-related deaths in women worldwide [1]. Ten to fifteen percent of breast cancers in Western women are triple-negative breast cancers (TNBCs), which do not express the estrogen receptor (ER), progesterone receptor (PR), or human epidermal growth factor receptor 2 (HER2) and have a poor overall prognosis and high recurrence risk [2]. TNBCs remain a global clinical management challenge due to their aggressive characteristics and lack of targeted therapies [3]. Chemotherapy is used to treat primary and metastatic diseases, and while targeted therapies, including immunotherapy, are slowly emerging to treat TNBCs in the West [3].

To characterize TNBC biology, multiple groups have defined TNBC subtypes in the West using gene expression or integrated molecular profiles [4], [5], [6]. Immune cell infiltration into tumors is now recognized as intrinsic to tumor biology and immunotherapy responses, so a more granular analysis of immunity in TNBC is required for prediction and prognostication, especially given recent data showing that a proportion of patients respond well to immunotherapy - atezolizumab in combination with nab-paclitaxel for advanced PD-L1^+^ TNBCs (IMPassion130 study) [7]. This is no longer an approved drug in the United States (US) after the outcomes from the follow-up trial – IMPassion131 study [8,9]. Moreover, pembrolizumab (anti-PD1 immunotherapy), combined with neoadjuvant chemotherapy, has been approved in the United States for treating high-risk early-stage TNBCs (based on KEYNOTE-522 study) [10]. However, the unaddressed question is whether TNBCs can be stratified into immune subtypes (immunotypes) that have distinct molecular and cellular presentations of the disease associated with acute or chronic inflammation leading to immunogenic cell death via damage-associated molecular patterns (DAMP) and T-cell receptor signaling. Further deciphering an association with clinical outcomes (prognosis, menopausal status and other covariates) and therapy responses using cross-cancer comparison may allow developing strategies to select patients according to prognosis or response to therapy to optimize patient management.

Hence, we sought to understand TNBC heterogeneity and identified three robust immunotypes based on consensus clustering (a machine-learning (ML) approach) of the expression of immune-related genes. Our analysis suggests avenues to rationally understand the immune and clinical landscapes to potentially translate and personalize successful immunotherapy opportunities to TNBC patients globally.

## Methods

### Patient samples, clinical characteristics and RNA isolation

TNBC patient samples were retrospectively collected as two independent cohorts from different tertiary cancer care hospitals in Bangalore, India. Fifty out of 92 TNBC samples from one hospital, St. John's Research Institute, were identified to be qualified for the study (Supplementary Fig. 1A). Similarly, 38 out of 48 TNBC samples were qualified for the study from Shri Shankara Cancer Hospital and Research Centre. Both the studies were approved by the respective institutional ethics review board and informed consent was obtained from the subjects.

Primary tumor tissue samples were collected at the time of surgery before treatment, fixed in buffered neutral formalin and processed as paraffin embedded blocks. Sections were cut from these blocks, stained with hematoxylin and eosin (H&E) and examined by a pathologist to confirm the presence of tumor. Immunohistochemistry for ER, PR and HER2 were done for determining receptor status by standard procedures [11]. Only formalin-fixed paraffin embedded (FFPE) blocks containing pre-treatment tissue with greater than 50% cancer epithelial cells were used for molecular analysis. All clinical, histopathological and demographic details were collected from the hospital medical records (Supplementary Fig. 1B). Radiologically recorded distant metastases or histologically confirmed local recurrence and death related to disease had been documented.

RNA from all FFPE specimens was extracted by the Trizol method according to the manufacturer's instructions and as described [12]. Briefly, 2 × 20 um sections were deparaffinized using xylene, washed in absolute ethanol and overnight digested with Proteinase K. Lysate was taken for extraction and RNA recovered from the aqueous phase.

### nCounter profiling and immunotype analysis

nCounter Immune profiling (NanoString Technologies) [13], subtype or clustering analysis [14] and the probabilistic principal component and covariate analysis (PPCCA)-based ML method [15] were performed as described in the respective and cited publications. A detailed description has been provided in Supplementary Methods.

### TIL Scoring

Assessment of the tumor infiltrating-lymphocyte (TILs) was done according to guidelines established by the International TIL working group [16]. Large areas of central necrosis or fibrosis are not included for evaluation, and tumor was focused only on tumor-stroma at low magnification. Only mononuclear infiltrate of lymphocytes and plasma cells were included and granulocytic infiltrate in areas of tumor necrosis was not included. Based on the percentage of stromal TILs present, they were grouped into mild (no or minimal immune cells), moderate (tumor with intermediate/heterogenous infiltrate) and dense (tumor with high immune infiltrate) infiltrates.

### Immunohistochemistry and scoring

Immunohistochemistry (IHC) was done for CD68 according to standard procedures as described in Supplementary Methods.

## Results

### TNBCs are heterogenous but can be divided into distinct immunotypes with different prognoses

**Fig. 1:**
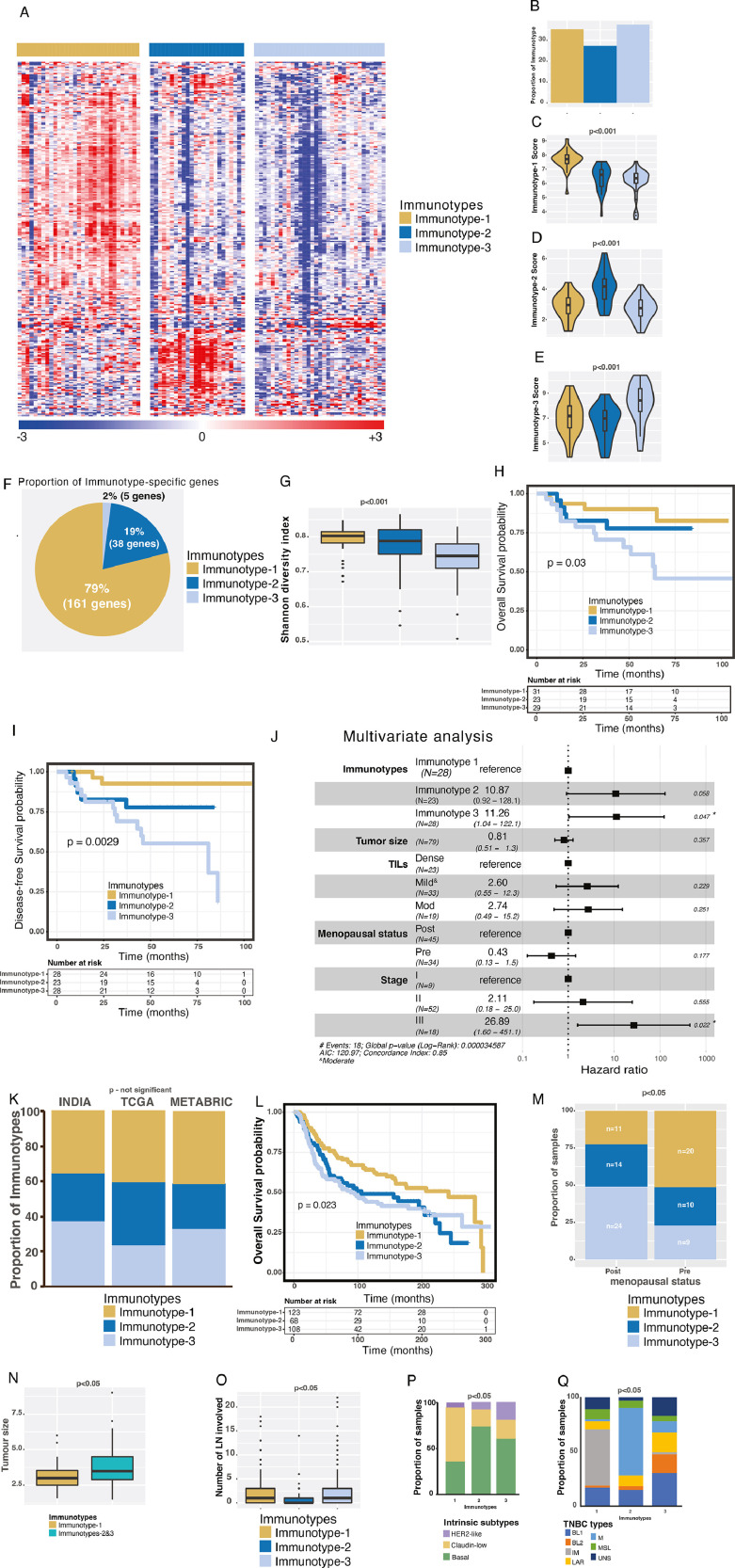
**Identification and clinical characterization of immune immunotypes using the Indian TNBC samples and comparison with Western TNBC cohorts. A.** Heatmap of immunotypes from Indian TNBC (*n*=88) identified by NMF clustering method. Immunotypes are shown on the top bar. The scales are shown at the bottom. **B.** Proportion of immunotypes from 88 Indian TNBCs. **C-E**. Immunotype-specific scores. **F.** Proportion of immunotype-specific genes out of 204 genes. **G.** Shannon diversity index of three immune immunotypes. **H-I.** Kaplan-Meier curves representing H) OS (*n*=83) and I) DFS (*n*=79) from the Indian TNBC cohort. **J.** Multivariate analysis of DFS and other clinical parameters. **K.** Proportion of three immune immunotypes in different cohorts of samples from India (*n*=88) and Western populations – TCGA (*n*=123) and METABRIC (*n*=299)**. L.** Kaplan-Meier curve representing OS from the METABRIC TNBC cohort (*n*=299). **M.** Proportion of pre- and post-menopausal samples represented in three immune immunotypes from the Indian TNBC cohort (*n*=81). **N.** Tumor size differences in immune immunotypes from the Indian TNBC cohort (n=86). **O.** Number of LNs involved in different immune immunotypes from the METABRIC cohort (*n*=299). **P-Q.** Proportion of P) intrinsic (*n*=284; 2 luminal-A and 10 normal-like samples were excluded), and Q) Vanderbilt immunotypes represented in immune immunotypes from the METABRIC cohort (*n*=268; see supplementary information). Kruskal-Wallis statistical test was performed for most of the analysis, except for survival analysis and proportion analysis, where the log-rank test was used for survival analysis and chi-squared test was used for the proportions. *p*-value of <0.05 was considered significant.

To characterize immune-based gene expression profiles of TNBCs in Indian women, 88 TNBC samples (Supplementary Figs. 1A-B and 2A-D) were profiled for 730 immune genes using the PanCancer Immune gene panel (NanoString Technologies), batch corrected (using exploBATCH ML method [15]) and normalised [13] (see Methods)). After selecting 392 variable genes (standard deviation ≥1), three distinct and robust TNBC immune gene expression immunotypes were defined using unsupervised nonnegative matrix factorization (NMF)-based consensus clustering (Fig. 1**A** and Supplementary Methods; Supplementary Fig. 2E-G and Supplementary Table 1A). The three immunotypes were present in approximately equal proportions (27-38%; Fig. 1**B**). Based on significance analysis of microarrays (SAM) and prediction analysis for microarrays (PAM), we derived 204 immunotype-specific (non-zero) highest PAM centroid scores (consolidated expression of genes across samples) from initial 281 genes (Fig. 1**C-F**; Supplementary Table 1B-D; see Supplementary Methods). A majority (79% and 19%) of 204 immunotype-specific genes belonged to Immunotype-1 and -2, respectively, whereas only 2% genes belonged to Immunotype-3 (Fig. 1**F**). Accordingly, Immunotype-1/-2 showed significantly (p<0.001) higher gene diversity as measured using the Shannon Entropy method, as previously described [17]; Fig. 1**G**), an index assessing immune gene expression patterns. Hence, Immunotype-1 shows immune-high gene expression patterns, whereas Immunotype-3 shows an immune-dormant or -exclusion pattern.

Immunotype-3 patients had significantly (p<0.05; log-rank test) poorer overall survival (OS) and disease-free survival (DFS; Fig. 1**H-I**) than Immunotype-1 (favorable prognosis) and Immunotype-2 (intermediate prognosis) patients. In a multivariate Cox regression model, Immunotype-3 (*p*=0.047) and stage III (*p*=0.022) were statistically significantly associated with DFS when controlling for clinical factors such as tumor size, TIL groups, and menopausal status (Fig. 1**J**).

Next, we investigated whether these immunotypes were present in TNBC samples from two Western cohorts - The Cancer Genome Atlas (TCGA, *n*=123) [18] and Molecular Taxonomy of Breast Cancer International Consortium (METABRIC; *n*=299) [19], which mainly represent Caucasian ethnic group. There was no significant difference in the distribution of immunotypes in the Indian TNBC cohort compared with the Western cohorts, suggesting that the Indian and Western TNBCs are similar (Fig. 1**K**, Supplementary Table 1E-F). There was also a similar trend in OS according to immunotype in the METABRIC data as in the Indian cohort (Fig. 1**L**). Hence, Indian and Western TNBC samples are considered similar throughout the manuscript.

### TNBC Immunotypes have distinct clinical and molecular subtype associations

Since the incidence of TNBC is reported to be higher in pre-menopausal women world-wide, including in India [2,20], we sought to further understand the association between TNBC immunotypes and menopausal status. Immunotype-1 TNBCs were more common in pre-menopausal patients and Immunotype-3 TNBCs were more common in post-menopausal patients (Fig. 1**M**; *p*<0.05). A similar, but not-significant, trend of menopausal status was observed in the TCGA (*p*<0.16) and METABRIC (p<0.28) data (Supplementary Fig. 3A-B). Related to these data and as expected, Immunotype-1 patients were younger (median age 44) than Immunotype-2 (median age 50) and Immunotype-3 (median age 54) patients (Supplementary Fig. 3C; *p*<0.05, Chi-square test), and this trend (not statistically significant) was similar in TCGA and METABRIC data (Supplementary Fig. 3D-E).

Interestingly, Immunotype-1 tumors were smaller than Immunotype-2 and -3 tumors (Fig. 1**N**), and Immunotypes-1 and -3 were associated with a greater number of involved LNs (from METABRIC data) than Immunotype-2 tumors (Fig. 1**O**). With respect to published intrinsic subtypes [21], Immunotype-1 shows a similar distribution of claudin-low and basal-like subtypes, whereas Immunotypes-2 and -3 predominantly show basal-like subtype. Immunotype-3 reveals increased HER2-like subtype, as assessed using METABRIC data (Fig. 1**P**). There was no significant association between METABRIC subtypes [19] and our immunotypes (Supplementary Fig. 3F). Interestingly, a majority of Immunotype-1 is associated with Vanderbilt's “immune” TNBC subtype [4] and Immunotype-2 with mesenchymal. In contrast, Immunotype-3 was a mixture of all Vanderbilt's subtypes (Fig. 1**Q**). Although TCGA immune subtypes [22] show a significant association with our immunotypes, there is no specific association of each immunotype with TCGA subtypes. Intriguingly, Immunotype-2 has more than 50% samples representing wounding healing C1 TCGA immune subtypes (Supplementary Fig. 3F). Overall, we identified three TNBC immunotypes with different immune gene expression, prognoses, and menopausal status.

### Immunotype-1 is enriched for Th1 cellular immunity and a cascade of immune changes

**Fig. 2:**
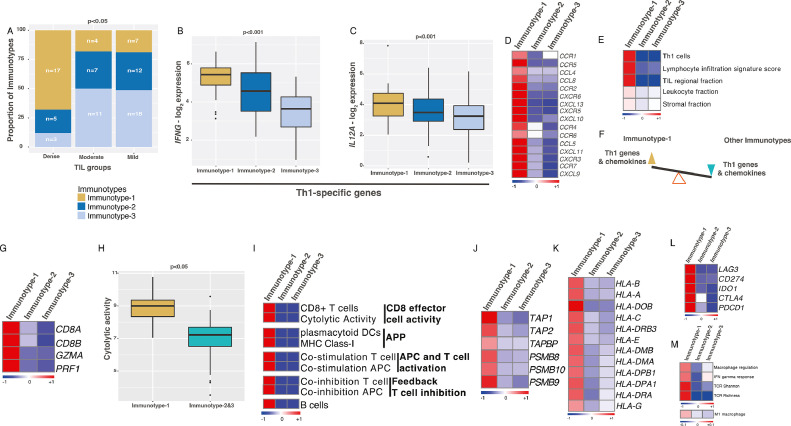
**Immune and clinical characteristics specific to Immunotype-1. A.** Proportion of dense, moderate, and mild TILS represented in three immunotypes from the Indian TNBC cohort (*n*=84). Chi-squared test was used for p-value calculation. **B-C.** Gene expression of Th1 response genes – (B) *IFNG* and (C) *IL12A* in immunotypes from the Indian TNBC cohort (*n*=88). **D.** Heatmap showing chemokine average expression per immunotype. **E.** Heatmap validation of specific immune characteristics (as mean values per subtype) in immunotypes from TCGA data (*n*=123). **F.** Schematic representing balance in Th1 response and chemokine gene expression in immunotypes. **G-L.** Heatmaps and boxplot showing changes in mean (G) CD8 T-cell-specific genes, (H) cytolytic activity, (I) T and B cell types and activities based on ssGSEA analysis, (J) antigen presenting and processing genes, (K) MHC-I &II HLA genes, (L) T-cell exhaustion genes per subtype from the Indian TNBC cohort (*n*=88). **M.** Heatmap showing macrophages, IFNG response, TCR diversity and intratumoral heterogeneity (as mean values per subtype) from TCGA data (*n*=123). All the figures used the Indian TNBC cohort (*n*=88), except figures (E) and (M), where TCGA data was used. Kruskal-Wallis statistical test was performed for p-value significance for those in boxplots.

We next investigated whether our immunotypes from the Indian cohort, representing the overall TNBC immune profiles, were associated with histological assessment of TILs. In our cohort, 44% had mild, 30% had dense, and 26% had moderate TILs. A majority of tumors with dense TILs were Immunotype-1 tumors (68%; *n*=25). However, a substantial (32%) of the Immunotype-1 tumors showed mild and moderate TILs. Hence, there is an opportunity beyond TIL scoring in stratifying TNBCs. On the other hand, those with moderate (82%; *n*=22) and mild (81%; *n*=37) TIL infiltrates were mainly Immunotype-2 and -3 tumors (Fig. 2**A**). With respect to immune gene and cellular composition, Immunotype-1 had higher expression of Th1-specific genes (*IFNG* and *IL12A*; Fig. 2**B-C**), reflecting increased cellular immunity. Interestingly, most chemokines represented in the 204 immunotype-specific genes were only highly expressed in Immunotype-1 (Fig. 2**D**). These patterns apparent in the Indian cohort were also seen in TCGA data, with increased Th1 cell, leukocyte, stromal, and TIL regional fractions and lymphocyte infiltration signature scores in Immunotype-1 (Fig. 2**E**). Hence, Immunotype-1 appears to have a gene expression profile balanced in favor of Th1 responses and chemokine expression (Fig. 2**F**).

We next explored the proportion and types of different immune cells within the three immunotypes using single-sample geneset enrichment analysis (ssGSEA) [23] and the entire 730 genes. The geneset distribution in the three immunotypes and the inferred cell populations significantly associated (FDR<0.2) with the three immunotypes are shown in Fig. 2**G-K**. Immunotype-1 showed increased CD8^+^ effector T-cells (and their genes *CD8A*/*CD8B*) and the cytolytic activity genes *GZMA and PRF1*, as assessed using cytolytic scores and ssGSEA (see Methods; Fig. 2**G-I**). This increase in CD8^+^ effector T-cell activity was also associated with patterns representing increased plasmacytoid dendritic cells, major histocompatibility class (MHC)-I, and co-stimulation of antigen presenting cells (APC) and T-cells and associated antigen processing and presentation (APP) genes, including genes representing transporter associated with antigen peptide transporters (TAP), immunoproteases, MHC class-II, and human leukocyte antigen (HLA) (Fig. 2**I-K**). Immunotype-1 also showed increased expression of immune checkpoint and T-cell exhaustion genes such as PD-1 (*PDCD1*), PD-L1 (*CD274*), *CTLA4*, and *LAG3* (Fig. 2**L**), suggesting that persistent stimulation of T-cells potentially promoted T-cell exhaustion through increased expression of these genes in Immunotype-1 tumors, as described [24]. Further interrogation of TCGA data showed increased INF-γ response genes and macrophage regulation, specifically anti-tumor M1 macrophages, in Immunotype-1 samples (Fig. 2**M**). We also examined T-cell receptor (TCR) and B cell receptor (BCR) repertoires in our immunotypes using TCGA data. As expected, TCR and BCR Shannon indices and richness scores were significantly higher in Immunotype-1 tumors, which might represent a highly reactive immune-associated stroma in the tumor (Fig. 2**E and M**). Our immunotypes capture an immune landscape and heterogeneity in TNBC beyond those represented by TIL grouping alone.

### Immunotype-1 immune changes are associated with DAMPs, acute inflammation, and viral-mimicry

**Fig. 3:**
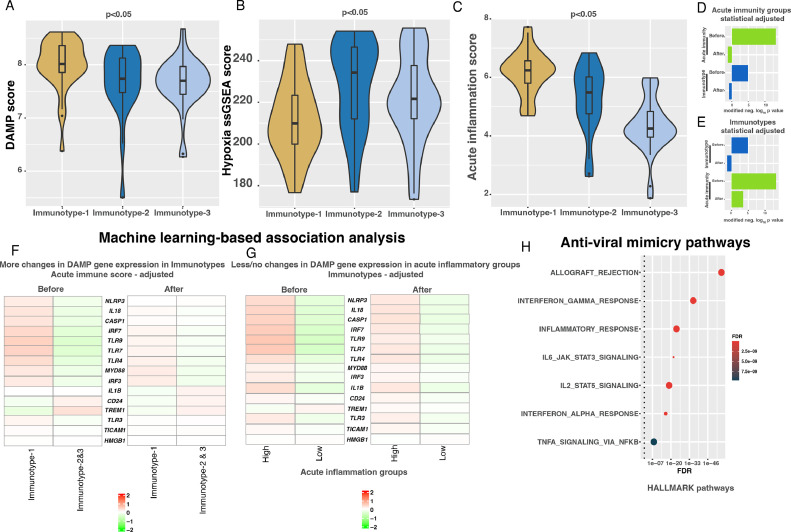
**Machine-learning based association analysis of acute inflammation, immunotypes and DAMP gene expression in the Indian cohort. A-C.** Boxplots showing differential changes in DAMP, hypoxia and acute inflammation scores from the Indian TNBC cohort (*n*=88). Kruskal-Wallis statistical test was performed for p-value significance. **D-E.** Barplots showing significant (*p*<0.01; linear regression) association of 15 DAMP genes with D) acute inflammation (high vs. low) and E) immunotypes (Immunotype-1 vs. others) in principal component (PC1) as assessed by PPCCA method using the Indian TNBC cohort (*n*=88). **F.** Heatmap showing change in 15 DAMP pathway genes (median expression across samples from each immunotype) in immunotypes before and after statistically adjusting acute inflammation using the *PPCCA* method using the Indian TNBC cohort (*n*=88). **G.** Heatmap showing change in 15 DAMP pathway genes (median expression across samples from each immunotype) in acute inflammation low and high groups before and after statistically adjusting immunotypes using the *PPCCA* method using the Indian TNBC cohort (*n*=88). **H.** Dotplot showing enrichment of anti-viral-mimicry pathways in Immunotype-1 from hallmarks gene sets from the Molecular Signature DataBase (MSigDB) database using Immunotype-1-specific genes. False discovery rate (FDR) was calculated from p-values from hypergeometric test using hypeR R package (see methods).

The cascade of adaptive immune responses and activation of dendritic cells is known to be associated with damage-associated molecular patterns (DAMPs), which are molecules released by acute inflammatory, hypoxic, or stressed cells linked to tumor progression or treatment [25]. Hence, we examined a score derived from the average of 15 genes representing various processes involved in DAMPs [26]. Interestingly, the DAMP gene score was significantly (*p*<0.05) higher in Immunotype-1 tumors compared with other immunotypes (Fig. 3**A**). Unlike DAMPs linked to hypoxia and adaptive immunity in our previous study of pancreatic neuroendocrine tumors [17], DAMPs were not linked to hypoxia in Immunotype-1 TNBCs, which had significantly lower hypoxia scores than Immunotypes-2 and -3 as assessed using ssGSEA and the hypoxia signature from MSigDB (Fig. 3**B**). Hence, we hypothesized that these significant increases in DAMP scores and Th1 responses are associated with acute inflammation in Immunotype-1 tumors. As predicted, the acute inflammation gene score (mean expression of *IL15, IL21*, and *LTA* in each sample) was significantly (*p*<0.05) higher in Immunotype-1 tumors compared with the other immunotypes (Fig. 3**C**).

To further explore if enrichment of DAMP genes is associated with (statistically dependent on) acute inflammation and Immunotype-1, we applied our previously described ML approach, PPCCA, [15] to the Indian TNBC cohort. This method combines probabilistic principal component and multivariate regression analyses to infer statistical associations or dependencies between three parameters (immunotypes, acute inflammation, and DAMP gene expression), which cannot be assessed by Pearson or other correlation analyses. For this purpose, we categorized the severity of acute inflammation into high and low based on the median acute inflammation score as a cut-off. Then, we removed the expression differences in 15 DAMP genes between the high and low acute inflammation samples by statistically adjusting or normalizing the expression data using PPCCA. This adjusted data led to the loss of association or dependency of Immunotype-1 on DAMP expression in TNBCs (p<0.05; modified negative log_10_ *p*-value > 0 in Fig. 3**D**), as assessed using the first probabilistic principal component (pPC1; with higher proportion of variability) and linear regression analysis with the PPCCA model. Before adjustment of acute inflammation by group, both acute inflammation groups and immunotypes were significantly (modified negative log_10_ p-value > 0 in Fig. 3**D**) associated with DAMP expression. This was also reflected as a partial change in DAMP gene expression between Immunotype-1 vs. Immunotypes-2 and -3 after adjusting the acute inflammation dependency in DAMP genes (Fig. 3**F**). Conversely, there was no/less loss of a significant association between acute inflammation and DAMP gene expression in pPC1 in the reverse analysis of adjusting immunotype dependency on DAMP gene expression, followed by regression analysis of acute inflammation groups in pPC1 (Fig. 3**E and G**). Moreover, we observed enrichment of host viral-infection mimicry pathways and genes in Immunotype-1 (Fig. 3**H**), in a similar way to our rectal cancer study. [27] These results demonstrate partial dependency of Immunotype-1 on acute inflammation for DAMP enrichment and provide clues that the progression of Immunotype-1 is related to viral-mimicry during infection.

### Immunotype-1-specific pathways and associated immunotherapies

**Fig. 4:**
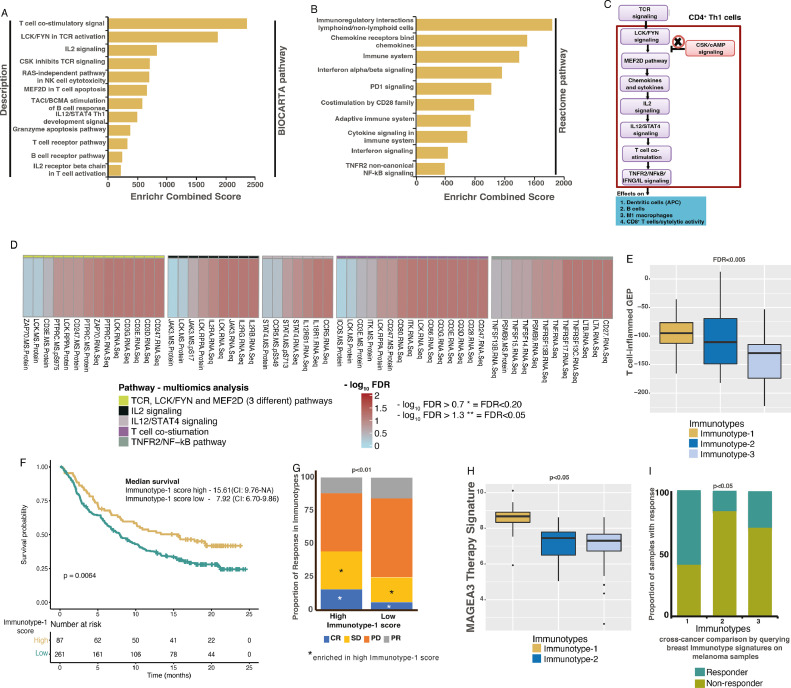
**Association of gene enrichment and therapy response to immune TNBC immunotypes. A-B.** Barplot showing gene enrichment of (A) BIOCARTA and (B) Reactome pathways using Enrichr tool (see Methods) in Immunotype-1. **C.** Schematic showing TCR and downstream signalling that effects immune cell types based on data curated from the Indian TNBC gene expression, pathway analysis in (A-B) and literature. **D.** Multiomics enrichment analysis validation specific pathways from (C) using TCGA TNBC samples (*n*=18). Kruskal-Wallis statistical test was performed for p-value significance. **E.** Boxplot showing differential T-cell-inflammed gene expression profile (GEP) in immunotypes in the Indian cohort (*n*=88). **F.** Kaplan-Meier curve and median survival data showing differential OS in melanoma samples (pre-treatment; Mariathasan et al. [36]; *n*=348) with high and low enrichment of Immunotype-1 genes and their association with immunotherapy response. Log-rank test was performed for p-value significance. **G.** Barplot showing the association of immune immunotypes with clinical RECIST response to immunotherapy in melanoma (Mariathasan et al. [36]; *n*=298). * - represents a significant (*p*<0.05; Kruskal-Wallis test) enrichment in samples with high Immunotype-1 score compared to those with low score. **H.** Boxplot showing differential MAGEA3 therapy response signature in immunotypes in the Indian cohort (*n*=88). **I.** Proportion of melanoma samples (*n*=56; GSE35640) showing immune TNBC immunotypes with differential MAGEA3 therapy response, as a cross-cancer comparison analysis. Kruskal-Wallis statistical test was performed for p-value significance for (E) and (H). Chi-squared test was performed for p-value calculations for (G) and (I).

Pathway analysis showed enrichment of Th1, IL2, IL12, TCR, BCR, cytolytic activity, PD-1, NFκB, chemokine/cytokine signaling and other related immune pathways in Immunotype-1 tumors using Indian samples (Fig. 4**A-B**; Supplementary Table 1G-H). These analyses allowed us to postulate a Immunotype-1-specific pathway in CD4^+^ Th1 lymphocytes (based on Fig. 4**A-B** and published studies [28,29]), where MEF2D pathway genes are activated through the TCR and LCK/FYN signaling (Fig. 4**C**). In this pathway, CD4^+^ T-cell co-stimulation, potentially via increased chemokines/cytokines and IL2, IL12/STAT4, and LCK/FYN signaling, further triggers TNFR2-specific NFκB signaling to increase interferon-γ signaling, connecting multiple studies [30], [31], [32], [33], [34]. To further validate this CD4^+^ Th1 lymphocyte signaling independently, we used TCGA multi-omics profiles, including RNA-seq-based gene expression and mass spectrometry- and reverse phase protein array (RPPA)-based protein expression, and associated them with our immunotypes. These independent analyses showed significant enrichment of all genes and majority of (total and/or phosphorylated) proteins from these representative pathways, suggesting activation of this pathway in Immunotype-1 samples (Fig. 4**D**).

Based on the above data and immune checkpoint gene expression (Fig. 2**L**), we hypothesized that Immunotype-1 tumors might be amenable to targeting with immune checkpoint inhibitors. Hence, we applied the expanded interferon-γ response gene signature from Ayers, et al. (also known as T-cell inflammed gene expression profile; GEP) to our data, which has been shown to be associated with responses to anti-PD-1 therapy in multiple cancer types [35]. We detected a significant association between the T-cell inflammed GEP and Immunotype-1, suggesting that Immunotype-1 may respond to anti-PD-1 immunotherapy (Fig. 4**E**). Moreover, we applied Immunotype-1 scores to Mariathasan et al., gene expression profiles, where melanoma patients were treated with anti-PD-L1 therapy [36]. Despite the difference in cancer types, we observed that melanoma patients with high Immunotype-1 scores had a better prognosis and enriched significantly (p<0.05) for complete response and stable disease than patients with low scores (Fig. 4**F-G**).

Similarly, a signature derived from MAGE-A3 vaccination therapy [37] was significantly associated with Immunotype-1 tumors (Fig. 4**H-I**) and, correspondingly, a majority of samples from melanoma patients responding to MAGE-A3 vaccination were significantly enriched for the Immunotype-1 signature. These results suggest mechanisms of immune evasion in Immunotype-1 breast tumors that may be targeted by anti-checkpoint therapy or MAGE-A3 vaccination, which warrants further assessment in TNBCs.

### Immunotype-2 is associated with Th2-based humoral and Th-17 immunity

**Fig. 5:**
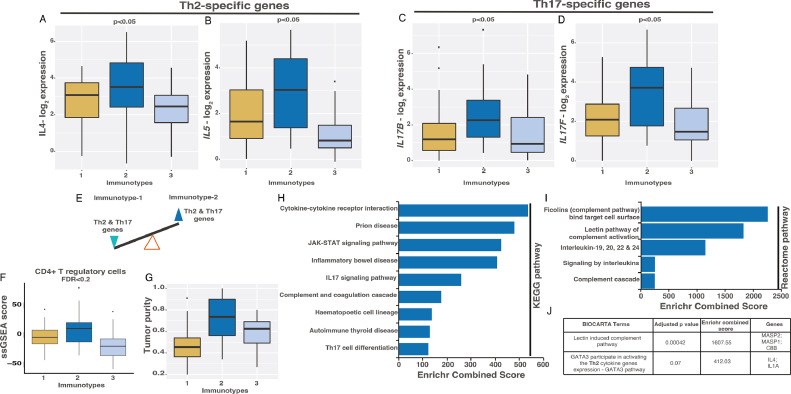
**Immune characteristics specific to Immunotype-2. A-D.** Gene expression of Th2 and Th17 response genes – (A) *IL4,* (B) *IL5*, (C) *IL17B* and (D) *IL17A* in immune immunotypes using the Indian TNBC cohort (*n*=88). **E.** Schematic representing balance in Th2 and Th17 response and chemokine gene expression in immunotypes. **F.** Boxplot showing changes in CD4+ T regulatory cells in immune immunotypes as assessed by ssGSEA analysis using the Indian TNBC cohort (*n*=88). **G.** Boxplot showing changes in tumor purity in immune immunotypes from the TCGA data (*n*=101). **H-I.** Barplot showing enrichment of (A) KEGG and (B) Reactome pathways using Enrichr tool (see Methods) in immunotype-2. **J.** A table showing BIOCARTA enrichment analysis in Immunotype-2. Kruskal-Wallis statistical test was performed for p-value significance for A-G).

In contrast to Immunotype-1, the characteristics of Immunotype-2 included Th2 and Th17 gene expression, representing a shift towards humoral and Th17 immunity (Fig. 5**A-E**). As expected, this Th2 response was associated with an increased proportion of CD4^+^ regulatory T cells in Immunotype-2 tumors (Fig. 5**F**). When the tumor cell fraction was evaluated using TCGA data, Immunotype-2 showed increased tumor purity (enriched for cancer cells rather than stroma) compared with the other two immunotypes, opposite to Immunotype-1 (Figs. 5**G** and 2**E**).

Next, we evaluated pathway regulation in Immunotype-2 in the Indian cohort. Immunotype-2 was enriched for cytokine signaling, specifically via IL17 (Th17) and lectin via the ficolin-based complement and coagulation cascade pathways (Fig. 5**H-J**, Supplementary Table 1I). Also, the data suggests a balance tipping towards GATA3-induced Th2 cytokines in this immunotype (Fig. 5**J**). Overall, the Th2 response in Immunotype-2 differs from Immunotype-1.

### Immunotype-3 is an immune desert subgroup but enriched for innate immune cells

**Fig. 6:**
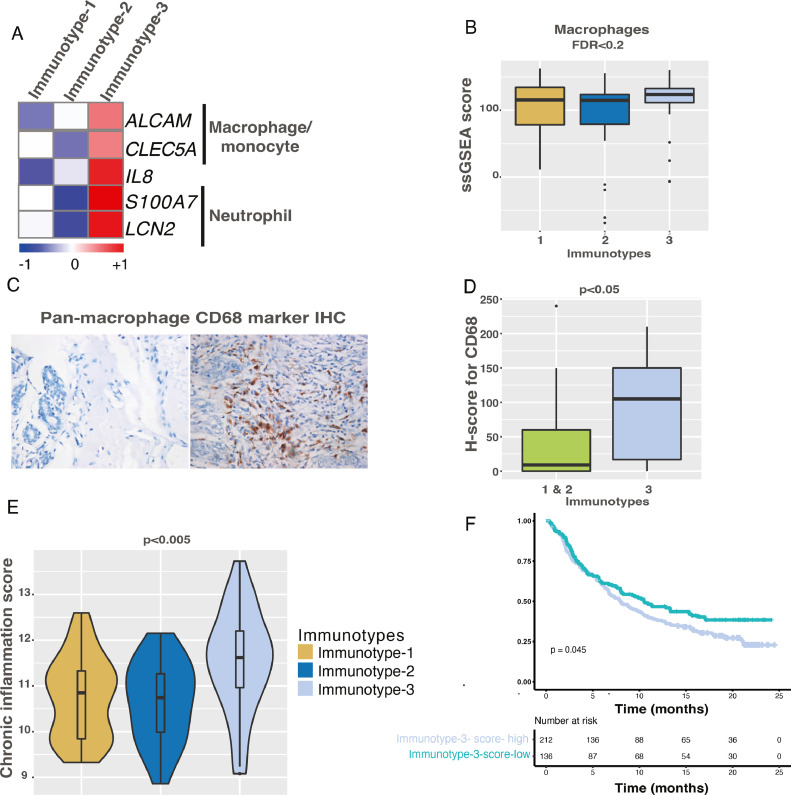
**Immune characteristics and validation of macrophage markers in Immunotype-3. A.** Heatmap showing Immunotype-3 specific genes associated with macrophages and neutrophils. **B.** Boxplot showing changes in macrophages in immune immunotypes as assessed by ssGSEA analysis using the Indian TNBC cohort (*n*=88). **C-D.** IHC and quantitation (n=39) of pan-macrophage marker – CD68 in immune immunotype samples using the Indian cohort. **E.** Boxplot showing differential changes in chronic inflammation scores in immune immunotypes using the Indian cohort (n=88). **F.** Kaplan-Meier curve and median survival data showing differential OS in samples with high and low enrichment of immune TNBC Immunotype-3 genes in melanoma samples (pre-treatment; Mariathasan et al. [36]; n=348). Kruskal-Wallis statistical test was performed for p-value significance for A), D) and E). Log-rank test was performed for p-value significance for F).

**Fig. 7:**
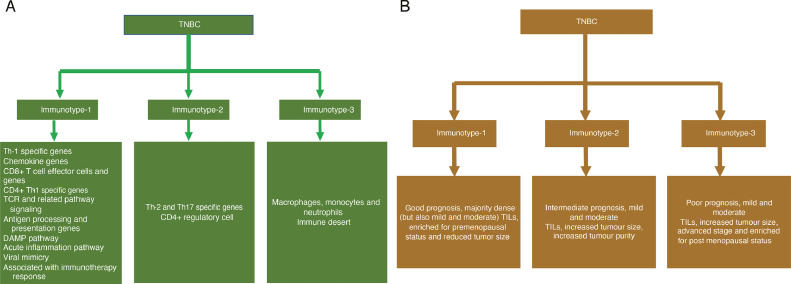
**Summary of TNBC immunotypes and their characteristics. A.** Molecular characteristics of immunotypes. **B.** Clinical characteristics of immunotypes.

Immunotype-3 samples significantly expressed only five immunotype-specific immune genes and therefore, represents an immune desert immunotype. However, there was high expression of five innate immune genes and cell types, specifically macrophage and neutrophil genes (Fig. 6**A-B**). Interestingly, Immunotype-3, along with Immunotype-2, was relatively hypoxic compared with Immunotype-1 (Fig. 3**B**). We confirmed the presence of macrophages using the pan-macrophage marker CD68 in immunotype-specific samples (*n*=39), which were indeed significantly more numerous in Immunotype-3 samples (Fig. 6**C-D**). CD68 positivity was confined to the tumor stroma and not epithelial nests. As opposed to Immunotype-1, there is an increased chronic inflammation signature in Immunotype-3 patients (Fig. 6**E**). The Immunotype-3 signature was associated with a worse prognosis (borderline significance) in melanoma patients in Mariathasan et al. dataset [36] (Fig. 6**F**). The overall summary of all the immunotypes is provided in Fig. 7**A-B**.

## Discussion

TNBC is increasingly recognized as a heterogeneous disease with high recurrence and mortality rates that more often displays aggressive clinical features such as high grade and lymph node positivity [2]. Despite international efforts to identify effective therapeutic targets in TNBCs by employing multi-omics strategies including proteomics, genomics, transcriptomics, and methylomics, only a handful of targeted therapies exist or under consideration for this subgroup [3]. Breast cancer was one of the first cancer types to undergo molecular classification. [21] Multiple groups have defined immunotypes in Western TNBCs [4], [5], [6], and here, using Indian samples, for the first time, we define three immune TNBC immunotypes with distinct gene and cell type profiles and prognoses (Fig. 7**A-B**). To avoid bias, we retrospectively analyzed two distinct cohorts of retrospective samples from two different Indian hospitals. Although these samples were collected from one major metropolitan city in South India (Bangalore), the clinical parameters were comparable to other Indian breast cancer studies, in that half of patients were aged 50 or under and over two-fifths were premenopausal [20,38]. The age and menopausal symptom characteristics more closely resembled an African and African-American populations but were distinct from those usually seen in Western populations [39,40]. There is an association between our's and Vanderbilt's subtypes, however, our immunotypes are specifically defined based on gene expression representing immune cell infiltrations that display different characteristics from Vanderbilt's immunotypes. These immunotypes defined in an Indian population were also present in Western TNBC samples and in approximately the same proportions. Nevertheless, the observed geographic differences are probably less to do with the intrinsic immune biology of TNBC.

Although the incidence of TNBC is higher in young, premenopausal women [38], most of these women with TNBC belonged to the good prognosis Immunotype-1 with active Th1 responses. In contrast, most post-menopausal women with TNBC had poor prognosis, immune desert, Immunotype-3 tumors. This increased risk of poor prognostic Immunotype-3 in this age group may be attributable to compromised immune function, specifically dysregulated adaptive Th1 responses, with aging. The capacity to develop memory T-cells against cancer antigens is reduced in older people, compromising the ability of the immune system to reject cancer cells [41]. Therefore, subsets of pre-menopausal and post-menopausal women with TNBC may require distinct immunotherapies depending on their immunotype.

Here, we have also attempted to understand the mechanistic insights of these immunotypes associated with different immune infiltrations. The cytolytic activity of CD8^+^ T-cells seen in Immunotype-1 depends on dedicated APCs, i.e., dendritic cells and, consistent with this, there was increased MHC-I expression and antigen processing and HLA genes in these tumors, which lead to co-stimulation of T-cell genes via TCR signaling in CD4 T-helper cells. Using pathway and literature [28], [29], [30], [31], [32], [33], [34] analysis, we found that Immunotype-1 was enriched for DAMP and CD4 T-helper TCR signaling, and we further validated TCR and related signaling pathways using multi-omic gene and protein data using TCGA profiles. This pathway may be associated with increased cytolytic activity in CD8^+^ T-cells, potentially reducing tumor progression and contributing to the better prognosis seen in patients with Immunotype-1 TNBCs. There was also increased expression of MHC-II-associated HLA genes representing potential activation of CD4, dendritic cells and M1 macrophages in Immunotype-1 tumors, which are known to be associated with a good prognosis in several tumor types [42]. In contrast, Immunotype-3, with its low proportion of M0 and M1 macrophages and higher proportion of M2 macrophages (validated using CD68 IHC) and neutrophils, was associated with poorer outcomes. This is consistent with a study by Gentles et al., who found that macrophages, neutrophils, and plasma cells are poor prognostic markers in both breast and lung cancer [43].

Collectively, our findings show that there is considerable immune infiltrate variability in TNBCs, which is partly determined by the molecular characteristics of the primary tumor and that influences clinical outcomes. For example, we found that Immunotype-1 may be primarily associated with an acute immune response, as described previously in other cancers [44]. Remarkably, using our PPCCA ML approach [15], we demonstrated a significant multivariate association between acute immunity and immunotypes with DAMP gene expression, suggesting increased acute immunity may be associated with or driving Th1-enriched Immunotype-1 TNBCs, a hypothesis that requires further validation. On the other hand, Immunotype-3 may be associated with chronic inflammation and hypoxia, leading to a poorer prognosis. While the roles of Th1 responses in acute inflammation and Th2 and M2 macrophages in chronic inflammation during cancer development are well described, [44] here we relate this to differential stratification, prognosis, signaling, and therapeutic responses. Our findings show that there is a complex relationship between acute/chronic immunity, intratumoral immune cell heterogeneity, molecular subgroup, and disease progression in TNBC. Treatments that aim to enhance Th1-based cellular immunity against tumors are only effective in a subset of patients [45], and our findings may help to identify this population using our Indian TNBC signature for specific targeting and further therapeutic development.

The success of immunotherapy in a subset of TNBC patients has raised hope of efficacy in TNBCs, [7,10] and TNBCs with dense TILs are expected to have a better prognosis [46]. Nevertheless, the objective response rate (ORR) for immune checkpoint inhibitor (ICI) monotherapy in metastatic TNBC is only modest, and the IMpassion130 trial for locally advanced and metastatic TNBC demonstrated that combined atezolizumab and nab-paclitaxel was more effective than monotherapy with nab-paclitaxel alone [7]. In contrast, a follow-up trial, IMPassion131 showed no improvement in patient survival with the same therapy [9]. Furthermore, several small studies have reported only marginal ORRs with ICIs, with poor responses likely to be multifactorial and include molecular heterogeneity, the use of ICI as monotherapy, and their use in non-first-line settings [3]. This is likely to be due, at least in part, to overall low PD-L1 expression (biomarker) in TNBCs, [47] so it is essential to develop robust biomarkers to select patients who respond to immunotherapy.

Our data suggest Immunotype-1 TNBC is potentially targetable with immunotherapy based on cross-cancer analysis using melanoma data. Specifically, Immunotype-1 was associated with immune checkpoint inhibitor (ICI) responses and prognosis, as assessed using the anti-PD-1 treatment prediction signature from Ayers et al. [35] and prognosis analysis using an anti-PD-L1 treated melanoma dataset [36].Our study also suggests an association between MAGEA3 immunotherapy and Immunotype-1 tumors. Recently, a phase-III trial assessing MAGEA3 immunotherapy in stage III melanoma patients in the adjuvant setting failed [48]. Although MAGEA3-positive cutaneous melanoma patients were selected for this trial, they were not further selected based on immune landscape such as those defined here. Similarly, a recent study has implicated the role of MAGEA3 in hepatocellular carcinoma (HCC) and suggested as a novel therapeutic avenue of targeting MAGEA3 for HCC [49]. In contrast, It is currently uncertain what therapy would best suit patients with Immunotype-2 tumors characterized by Th2/Th17 responses, infrequent LN involvement, an intermediate prognosis, and increased tumor purity; further investigation is required to identify potential immunotherapy targets associated with the immune complement cascade and GATA3. The increased wound healing response in Immunotype-2 is congruent with the association of Th2 response with wound healing [50], which might qualify them for antiangiogenic immunotherapies as described [51]. However, this immunotype may benefit from bioengineered immunotherapy using collagen-binding domain (CBD)-IL12 to reactivate Th1 pathways, [52] followed by treatment with an ICI. Immunotype-3 was also associated with increased poor prognostic basal-like and HER2-like intrinsic immunotypes compared with Immunotype-1. Based on our data, Immunotype-3 may not respond to first-line ICI-based immunotherapy.

## Conclusion

Here we characterized the immune heterogeneity in TNBCs in Indian women. In doing so, we identified three immunotypes with distinct immune cell infiltrates, immune cell signaling, and gene signatures associated with prognosis and responses to immunotherapy. Overall, immune gene expression in Indian and Western TNBCs appears to be largely similar. This immune-transcriptome study suggests that therapies targeting immune microenvironment in Western populations may be as effective in Indian populations, however, genetic (DNA-based) changes may need to be considered for geography-specific personalized therapy. This may accelerate pharmaceutical adoption to Indian TNBC patients.

## Ethical approval and consent to participate

This study involves retrospective analysis of samples and was approved by the respective institutional ethics review board. Informed consent was obtained from the subjects.

## Funding

We thank Newton Fund and Global Challenge Research Fund (GCRF)-based funding provided through Research England to the ICR, which partly supported this work related to molecular profiling and data analysis. We thank Nadathur Holdings Pvt Ltd and Bagaria Trust whose generous funding supported patient recruitment, clinical follow up and analysis.

## Authors’ Contributions

AS conceived and developed the idea; AS and AK further developed this idea; AK, JP, SA, RBD, and SBS provided patient samples and clinical follow up data; AS, AK, HPS, CR, YP, and KD performed the experiments; AK and JP performed histopathology and related data analysis; AMa, AMe and MC critically read the manuscript; AS, AK and HPS wrote the manuscript; AS, MC and AK supervised the study.

## Declaration of Competing Interest

AS has the following patents - Patient classification and prognostic method (GEP-NET) – Priority Patent – EP18425009.0, Patent – “Molecular predictors of therapeutic response to specific anti-cancer agents” (patent number US9506926B2). AS serves as a Scientific Advisor for Diagnostring Laboratories and Enedra Therapeutics and served as an Advisor for 4baseCare.

## Appendix Supplementary materials

Image, application 1

Image, application 2

Image, application 3

Image, application 4

Image, application 5
