# Supplementary material for: Comprehensive characterization of immune landscape of Indian and Western triple negative breast cancers
Source: Transl Oncol. 2022 Aug 11;25:101511. doi: 10.1016/j.tranon.2022.101511 (PMC9386467; doi:10.1016/j.tranon.2022.101511)
Supplement: Supplementary file 2 [file mmc2.pdf]

Supplementary Figure 1

A

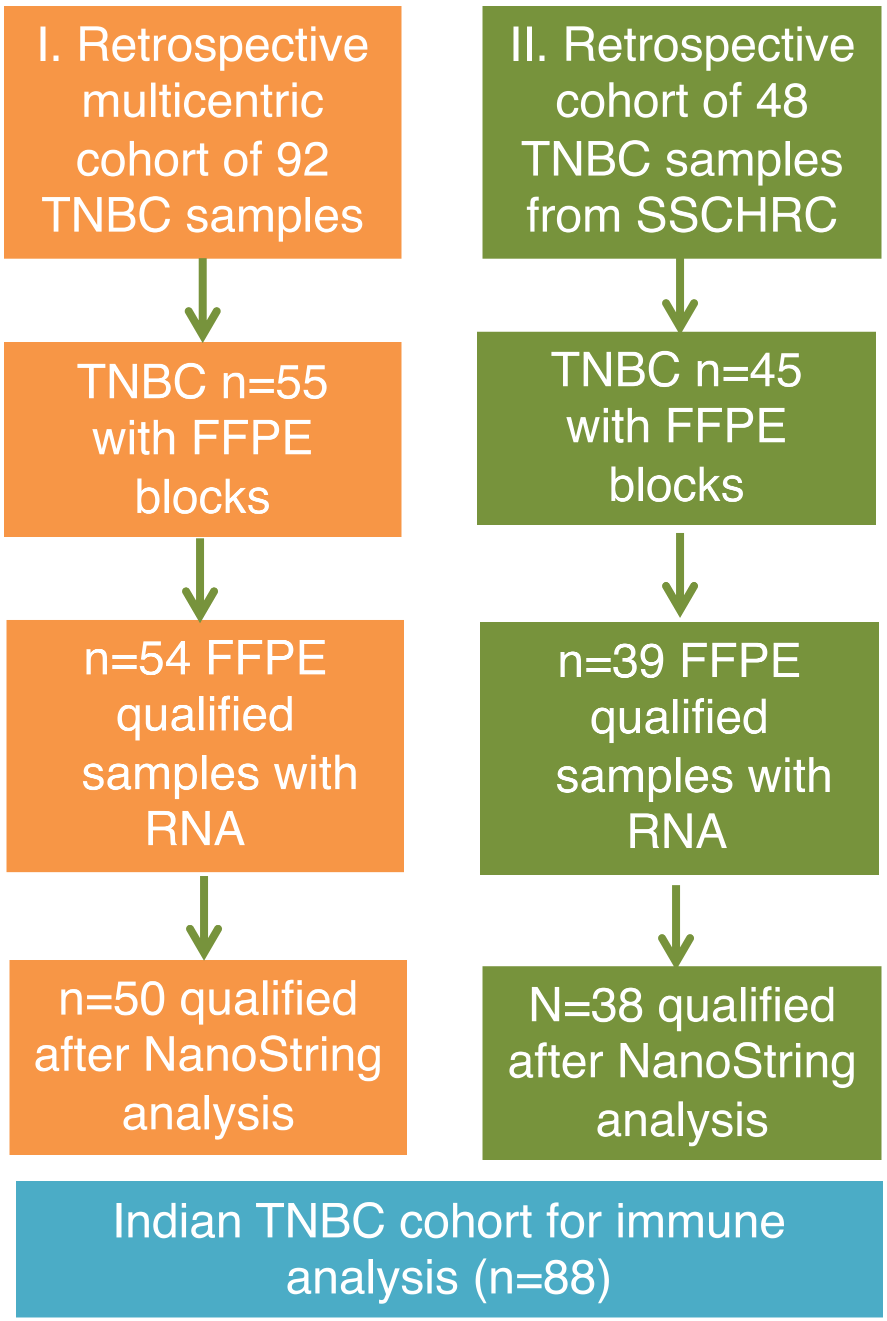


B

| Clinical Table(N=88) | | N(%) |
|---|---|---|
| Age(yrs) | Mean | 50 |
| | Median | 51 |
| | ≤50 | 44(50) |
| | >50 | 44(50) |
| Menopausal status | Pre | 39(44) |
| | Post | 49(56) |
| Grade | 2 | 17(19.3) |
| | 3 | 68(77.3) |
| | NA | 3(3.4) |
| Tumor Size | <2 cm | 7(8) |
| | 2-5 cm | 68(77.2) |
| | >5 cm | 11(12.5) |
| | NA | 2(2.3) |
| Stage | I | 9(10) |
| | II | 59(67) |
| | III | 20(23) |
| Lymph node status | Positive | 33(38) |
| | Negative | 53(60) |
| | NA | 2(2) |
| Lymphocyte infiltrate groups | Dense | 25(28) |
| | Mild | 37(42) |
| | Moderate | 22(25) |
| | NA | 4(5) |
