# Supplementary figures and images for: Comprehensive characterization of immune landscape of Indian and Western triple negative breast cancers
Source: Transl Oncol. 2022 Aug 11;25:101511. doi: 10.1016/j.tranon.2022.101511 (PMC9386467; doi:10.1016/j.tranon.2022.101511)

Supplementary Figure 3

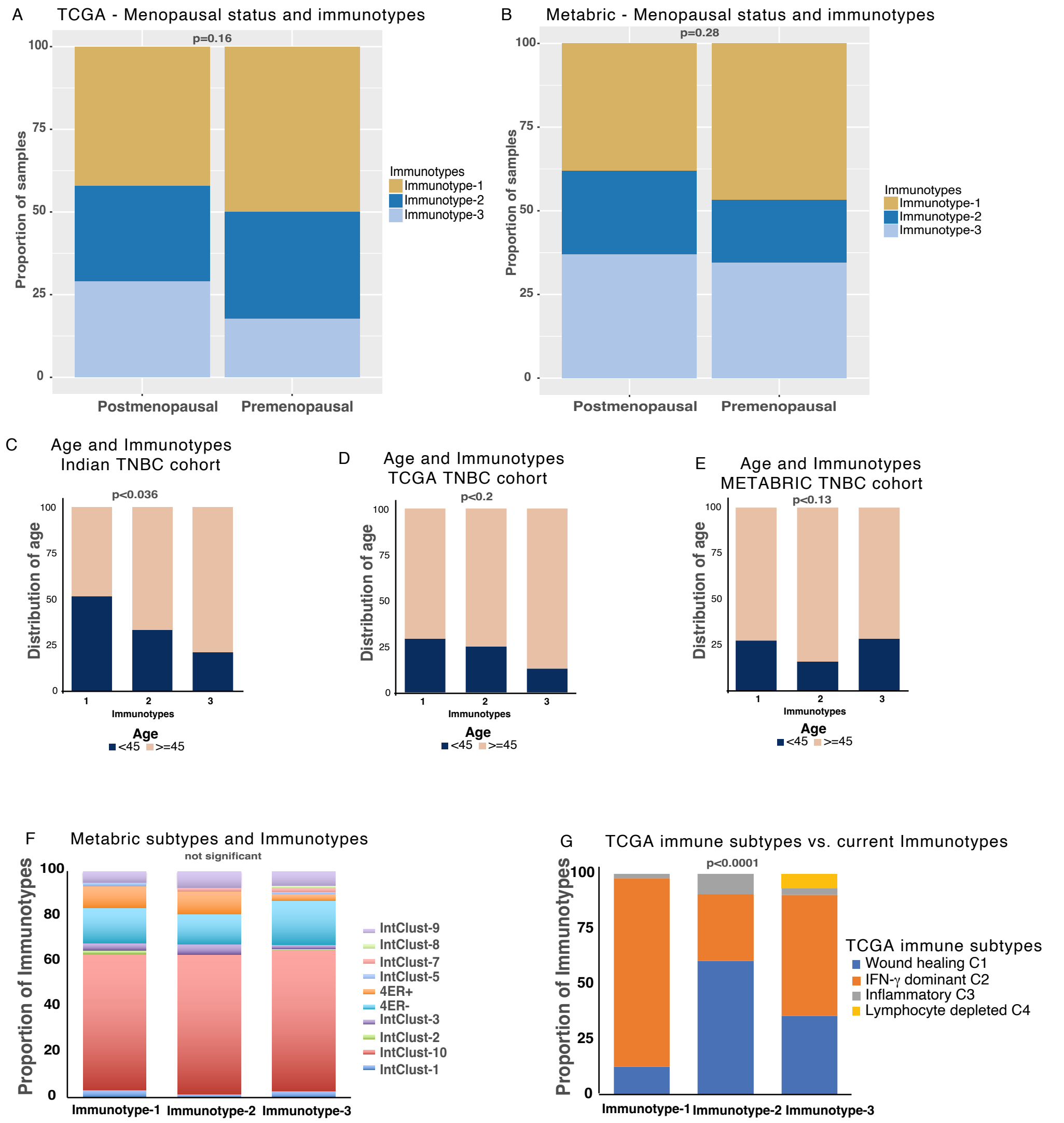

Supplement: Supplementary file 4 [file mmc4.pdf]
