# Supplementary material for: Comprehensive characterization of immune landscape of Indian and Western triple negative breast cancers
Source: Transl Oncol. 2022 Aug 11;25:101511. doi: 10.1016/j.tranon.2022.101511 (PMC9386467; doi:10.1016/j.tranon.2022.101511)
Supplement: Supplementary file 5 [file mmc5.docx]

**Supplementary Information**

**Comprehensive characterization of immune landscape of Indian and Western triple negative breast cancers**

**Supplementary Methods**

**Assessment of clinical characteristics**

The prognostic value of the immunotypes was assessed using Kaplan-Meier curve, log rank test using overall survival (OS) and disease-free survival (DFS) and multivariable Cox regression using DFS.

**nCounter Immune Profiling, batch effect and variable gene analysis**

Immune gene profiling using the NanoString Technologies’ nCounter® PanCancer Immune Profiling Panel and data analysis using nSolver software was carried out as per the manufacturer’s instructions and as described.^1^ The immune profiling for 88 Indian TNBC samples was performed in two batches based on the sources of the samples. Only those 693 genes (out of 730 genes) with zeros greater than or equal to 80% of the samples were considered. Diagnosis and correction of batch effects of the immune profiles were performed using the exploBatch machine-learning (ML) tool.^2^ Highly variable 392 genes with SD>1 was selected for further analysis.

**Other datasets and subtypes**

For validation and comparison purpose, the publicly available Western cohort of only TNBC samples from TCGA^3^ (123 TNBC samples; data downloaded from the Xena platform^4^ in 2019) and METABRIC^5^ (299 TNBC samples; data downloaded from cBio cancer genomics portal^6^) data, along with their clinical and molecular characteristics were used. In addition, multiomics data from TCGA^7^ were used for immunotype-specific signaling pathway analysis. For immunotherapy treatment assessment, Marthiasan et al.,^8^ (n=348) gene expression and clinical dataset was used. To assess MAGEA3-based therapy, Ulloa-Montoya, et al.,^9^ (n=65), gene expression data (GSE35640) and therapy response was used. Vanderbilt et al.,^10^ subtypes for METABRIC^5^ were assessed using TNBCtype tool^11^ (the tool removed 31 samples out of 299 TNBC samples by IHC as enriched for ER gene expression, whereas these 31 samples were ER negative by METABRIC IHC).

**NMF and SAM/PAM (subtype/immunotype) Analysis**

NMF-based unsupervised clustering analysis was performed to identify immunotypes by applying the *NMF* R package and Lee and Seung method.^12-14^ The optimal number of immunotypes were determined based on cophenetic co-efficient and Silhouette analysis of NMF clusters, as described^15-17^. SAM and PAM analyses were performed using the *siggenes* and *pamr* R libraries, respectively, as described.^18,19^

**Immune cell, activity, pathway and analysis**

Cytolytic activity was calculated as a geometric mean of *GZMA* and *PRF1* genes as described previously^20^. Immune cell analysis was performed using ssGSEA^21^ and Rooney et al.,^20^ gene signatures for immune cell types. T cell-inflammed GEP was calculated as a mean expression of 18 genes from Ayer’s et al.^22^ KEGG^23^ and REACTOME^24^ pathway analyses was performed using Enrichr.^25^ Acute (*IL15*, *IL21* and *LTA*) and chronic (*VEGFA*, *MIF* and *S100A8*) inflammation, DAMP (15 genes from Tang et al.^26^) and hypoxia (from Molecular Signature Database; MSigDB^27^) gene scores were calculated as mean expression per sample.

**PPCCA and multivariate gene and covariate analysis**

The PPCCA method^2^ was applied to assess the association between acute inflammation score, immunotypes and DAMP gene expression data, as described previously by us.^28^ The tool is available at <https://github.com/syspremed/exploBATCH>.

**Immunohistochemistry and scoring**

IHC was done for CD68 according to standard procedures. Briefly, sections (5µ in thickness) were cut from FFPE blocks on poly L-lysine coated slides and subjected to deparaffinization in xylene and rehydrated in graded alcohol. After blocking endogenous peroxidase with a 3% hydrogen peroxide solution, antigen retrieval was done in 0.01M EDTA buffer at pH 8, in a heat triggered multi epitope retrieval system for immunohistochemistry from PathinSitu (Cat # MPS001). Primary blocking was done with 3% bovine serum albumin (BSA, Sigma) for 30 min at room temperature. Primary antibody for CD68(Clone KP1, Mouse monoclonal, Dako, IS609) 1 hr at room temperature. Sections were further incubated with secondary antibody (DAKO REAL^™^EnVision^™^) for 30 min as per the kit instructions, followed by development of the colour using DAB (DAKO REAL^TM^EnVision^TM^) for 10 min. Sections were counterstained with hematoxylin and mounted after dehydration in graded alcohol and xylene. Appropriate positive and negative controls were run for each batch. Microscopy and scoring of CD68 was done to record intensity of staining, proportion of the cells stained and localization of the protein.

**Supplementary Figure Legends**

**Supplementary Figure 1. Consolidated Standards of Reporting Trials (CONSORT) diagram and clinical characteristics of Indian TNBC samples. A.** Consort diagram showing workflow of samples selected for the study. **B.** A table of clinical characteristics of samples.

**Supplementary Figure 2. Batch diagnosis and correction and NMF analysis of 88 Indian TNBC samples.** **A-B.** Batch quantitative diagnosis using exploBATCH tool A) before and B) after batch correction of 88 Indian TNBC samples**. C-D.** Batch visual diagnosis using PCA C) before and D) after batch correction of 88 Indian TNBC samples. **E-G.** NMF analysis showing E) cophenetic analysis, F) Silhouette statistics and G) consensus plot of 88 Indian TNBC samples.

**Supplementary Figure 3. Menopausal, age and immunotype association analysis of TNBC samples. A-B.** Association of menopausal status with immunotypes in A) TCGA and B) METABRIC TNBC cohorts. **C-E.** Association of age with immunotypes in C) Indian, D) TCGA and F) METABRIC TNBC cohorts. **F-G.** Association of immunotypes with F) integrated breast cancer subtypes and G) TCGA immune subtypes.

**Supplementary Table Legends**

**Supplementary Table 1. A.** Indian TNBC samples and their immunotype identities. **B.** SAM analysis results. **C.** PAM Centroids. **D.** Immunotype specific genes. **E.** TCGA TNBC samples and their immunotype identities. **F.** METABRIC TNBC samples and their immunotype identities. **G.** BIOCARTA Immunotype-1 pathways. **H.** Reactome Immunotype-1 pathways. **I.** Reactome Immunotype-2 pathways.

References

1 Ragulan, C. *et al.* Analytical Validation of Multiplex Biomarker Assay to Stratify Colorectal Cancer into Molecular Subtypes. *Sci Rep* **9**, 7665, doi:10.1038/s41598-019-43492-0 (2019).

2 Nyamundanda, G., Poudel, P., Patil, Y. & Sadanandam, A. A Novel Statistical Method to Diagnose, Quantify and Correct Batch Effects in Genomic Studies. *Sci Rep* **7**, 10849, doi:10.1038/s41598-017-11110-6 (2017).

3 Ciriello, G. *et al.* Comprehensive Molecular Portraits of Invasive Lobular Breast Cancer. *Cell* **163**, 506-519, doi:10.1016/j.cell.2015.09.033 (2015).

4 Goldman, M. J. *et al.* Visualizing and interpreting cancer genomics data via the Xena platform. *Nat Biotechnol* **38**, 675-678, doi:10.1038/s41587-020-0546-8 (2020).

5 Curtis, C. *et al.* The genomic and transcriptomic architecture of 2,000 breast tumours reveals novel subgroups. *Nature* **486**, 346-352, doi:10.1038/nature10983 (2012).

6 Cerami, E. *et al.* The cBio cancer genomics portal: an open platform for exploring multidimensional cancer genomics data. *Cancer Discov* **2**, 401-404, doi:10.1158/2159-8290.CD-12-0095 (2012).

7 Mertins, P. *et al.* Proteogenomics connects somatic mutations to signalling in breast cancer. *Nature* **534**, 55-62, doi:10.1038/nature18003 (2016).

8 Mariathasan, S. *et al.* TGFbeta attenuates tumour response to PD-L1 blockade by contributing to exclusion of T cells. *Nature* **554**, 544-548, doi:10.1038/nature25501 (2018).

9 Ulloa-Montoya, F. *et al.* Predictive gene signature in MAGE-A3 antigen-specific cancer immunotherapy. *J Clin Oncol* **31**, 2388-2395, doi:10.1200/JCO.2012.44.3762 (2013).

10 Lehmann, B. D. *et al.* Identification of human triple-negative breast cancer subtypes and preclinical models for selection of targeted therapies. *J Clin Invest* **121**, 2750-2767, doi:10.1172/JCI45014 (2011).

11 Chen, X. *et al.* TNBCtype: A Subtyping Tool for Triple-Negative Breast Cancer. *Cancer Inform* **11**, 147-156, doi:10.4137/CIN.S9983 (2012).

12 Gaujoux, R. & Seoighe, C. A flexible R package for nonnegative matrix factorization. *BMC Bioinformatics* **11**, 367, doi:10.1186/1471-2105-11-367 (2010).

13 Brunet, J.-P., Tamayo, P., Golub, T. R. & Mesirov, J. P. Metagenes and molecular pattern discovery using matrix factorization. *Proceedings of the National Academy of Sciences* **101**, 4164-4169, doi:10.1073/pnas.0308531101 (2004).

14 Lee, D. D. & Seung, H. S. Learning the parts of objects by non-negative matrix factorization. *Nature* **401**, 788-791 (1999).

15 Collisson, E. A. *et al.* Subtypes of pancreatic ductal adenocarcinoma and their differing responses to therapy. *Nature Medicine* **17**, 500-503 (2011).

16 Sadanandam, A. *et al.* A cross-species analysis in pancreatic neuroendocrine tumors reveals molecular subtypes with distinctive clinical, metastatic, developmental, and metabolic characteristics. *Cancer Discovery* **5**, 1296-1313, doi:10.1158/2159-8290.CD-15-0068 (2015).

17 Sadanandam, A. *et al.* A colorectal cancer classification system that associates cellular phenotype and responses to therapy. *Nature Medicine* **19**, 619-625, doi:10.1038/nm.3175 (2013).

18 Tusher, V. G., Tibshirani, R. & Chu, G. Significance analysis of microarrays applied to the ionizing radiation response. *Proceedings of the National Academy of Sciences* **98**, 5116-5121 (2001).

19 Tibshirani, R., Hastie, T., Narasimhan, B. & Chu, G. Diagnosis of multiple cancer types by shrunken centroids of gene expression. *Proceedings of the National Academy of Sciences* **99**, 6567-6572 (2002).

20 Rooney, M. S., Shukla, S. A., Wu, C. J., Getz, G. & Hacohen, N. Molecular and genetic properties of tumors associated with local immune cytolytic activity. *Cell* **160**, 48-61, doi:10.1016/j.cell.2014.12.033 (2015).

21 Subramanian, A. *et al.* Gene set enrichment analysis: a knowledge-based approach for interpreting genome-wide expression profiles. *Proceedings of the National Academy of Sciences* **102**, 15545-15550 (2005).

22 Ayers, M. *et al.* IFN-gamma-related mRNA profile predicts clinical response to PD-1 blockade. *J Clin Invest* **127**, 2930-2940, doi:10.1172/JCI91190 (2017).

23 Kanehisa, M. & Goto, S. KEGG: kyoto encyclopedia of genes and genomes. *Nucleic Acids Res* **28**, 27-30, doi:10.1093/nar/28.1.27 (2000).

24 Wu, G. & Haw, R. Functional Interaction Network Construction and Analysis for Disease Discovery. *Methods Mol Biol* **1558**, 235-253, doi:10.1007/978-1-4939-6783-4_11 (2017).

25 Chen, E. Y. *et al.* Enrichr: interactive and collaborative HTML5 gene list enrichment analysis tool. *BMC Bioinformatics* **14**, 128, doi:10.1186/1471-2105-14-128 (2013).

26 Tang, D., Kang, R., Coyne, C. B., Zeh, H. J. & Lotze, M. T. PAMPs and DAMPs: signal 0s that spur autophagy and immunity. *Immunol Rev* **249**, 158-175, doi:10.1111/j.1600-065X.2012.01146.x (2012).

27 Liberzon, A. *et al.* The Molecular Signatures Database (MSigDB) hallmark gene set collection. *Cell Syst* **1**, 417-425, doi:10.1016/j.cels.2015.12.004 (2015).

28 Young, K. *et al.* Immune landscape, evolution, hypoxia-mediated viral mimicry pathways and therapeutic potential in molecular subtypes of pancreatic neuroendocrine tumours. *Gut*, doi:10.1136/gutjnl-2020-321016 (2020).
